# Study on the Cocrystallization Mechanism of CL-20/HMX in a Propellant Aging Process through Theoretical Calculations and Experiments

**DOI:** 10.1021/acsomega.1c07328

**Published:** 2022-02-18

**Authors:** Xitong Zhao, Xiaolong Fu, Guanglong Zhang, Xiangyang Liu, Xuezhong Fan

**Affiliations:** † Xi’an Modern Chemistry Research Institute, Xi’an 710065, Shaanxi, China; ‡ School of Aerospace Engineering, Beijing Institute of Technology, Beijing 100081, Beijing, China

## Abstract

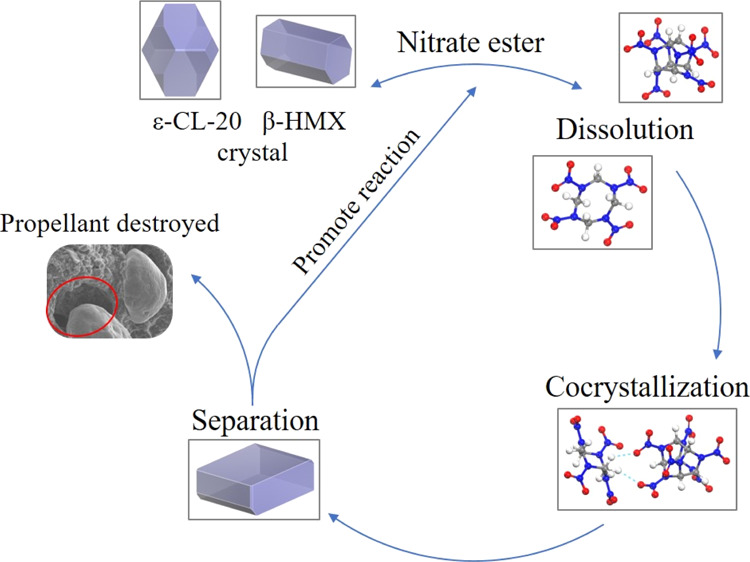

Energetic materials undergo physical and chemical aging due to environmental effects, resulting in the degradation of safety and detonation performances. Therefore, studying the aging performance of energetic materials is of great importance for the efficient application of 2,4,6,8,10,12-hexanitro-2,4,6,8,10,12-hexaazaisowurtzitane (CL-20)-based solid propellants. In this paper, XRD and FTIR of the CL-20-based propellant and CL-20/1,3,5,7-tetranitro-1,3,5,7-tetrazocane (HMX)-based propellant samples showed CL-20/HMX cocrystal formation according to appearance of new peaks. SEM and EDS analyses showed that pores and dehumidification in the propellant occurred with the cocrystallization of CL-20 and HMX during the aging process. Furthermore, molecular dynamics simulation was used to predict the crystal transformation of the CL-20- and HMX-based propellant under a long-term storage process. The stability of ε-CL-20 was obtained by analyzing the crystal transformation rate. The binding energy, radial distribution function between CL-20 and HMX, as well as mechanical properties of the CL-20/HMX cocrystal and the mixture were calculated to reveal the stronger binding between CL-20 and HMX in the cocrystal. Meanwhile, the inducer effect of a nitrate ester during the cocrystallization process was analyzed. The theoretical calculation shows that during aging, ε-CL-20 tends to exist stably, while CL-20/HMX tends to form cocrystals because of the strong bond. The present work on the transformation and cocrystallization of CL-20 and HMX during long-term storage is beneficial for understanding the degradation mechanism of the propellant performances, facilitating safe storage and life evaluation of propellants.

## 1 Introduction

Owing to the high density and energy, 2,4,6,8,10,12-hexanitro-2,4,6,8,10,12-hexaazaisowurtzitane (CL-20) has been respected as one of the most promising explosives with a superior comprehensive performance.^1,2^ The density, detonation velocity, detonation pressure, and energy of CL-20 are all greater than that of 1,3,5,7-tetranitro-1,3,5,7-tetrazocane (HMX).^3^ If a proportion of HMX in the high-energy propellants was to be replaced by ε-CL-20, the energy density of the propellants would be greatly enhanced. Considering that the cost of CL-20 is much higher and the yield is much lower than those of HMX, it would be ideal to replace part of HMX by CL-20 to improve the properties of the propellants.^4^ However, it will bring new aging problems with introducing CL-20 in the propellants, such as crystal phase transformation of CL-20 and cocrystallization of CL-20 and HMX.

CL-20 possesses four crystalline phases (α, β, γ, and ε) at room temperature, which present different density, sensitivity, detonation and thermodynamic stability.^1^ The transition energy barrier among these four different phases is low, which makes CL-20 prone to undesired crystal phase change.^5^ For the CL-20-based propellant, temperature has great impact on the CL-20 crystal phase. Although, ε-CL-20 can remain stable below 64 °C, it will transform to the γ-phase when the temperature increases to 74 °C.^6^ Millar et al. observed that ε-CL-20 can easily change to the α or γ crystalline phase at a high pressure and a low temperature.^7^ Therefore, improving the purity of the ε-phase and promoting its stability are important for developing CL-20-based energetic systems.

In recent years, CL-20-based cocrystals have been researched as a promising strategy to solve the high-sensitivity problem^8−10^ through the method of experiments^11−20^ and simulations.^21−25^ Due to the excellent properties of HMX and its wide application in propellants,^26,27^ the CL-20/HMX cocrystal has attracted the attention of researchers. Bolton et al. reported the cocrystal of CL-20 and HMX, exhibiting a plate habit, and noted that there are CH···O type of hydrogen bonds between CL-20 and HMX molecules.^28^ The theoretical detonation velocity of the obtained CL-20/HMX cocrystal (9484 m·s^–1^) is higher than that of β-HMX, and the shock sensitivity decreased to a value similar to that of β-HMX. This laid the foundation for the study of CL-20/HMX cocrystal energetic materials. However, cocrystallization of CL-20 and HMX during propellants’ aging process will bring negative effects. Cao et al. carried out a high-temperature accelerated aging test of the glycidyl azide polymer (GAP) high-energy propellant containing CL-20/HMX.^29^ During this process, the modulus of the propellant increases significantly, the elongation decreases rapidly, and the density decreases and the burning rate increases, which are typical aging properties.^30,31^ Through the physical and chemical analyses, they attributed the variations of the propellant to the dissolution and cocrystallization of CL-20 and HMX under the effect of a solvent.^32^ To date, the variation of the CL-20/HMX cocrystal and the evolution of intermolecular interactions between them in the propellant during aging have not been investigated systemically.

In a high-energy propellant, CL-20 and HMX can dissolve in a nitrate ester, and the critical transition temperature of CL-20 is decreased.^33,34^ Thus, it is worthy to study the mechanism of the CL-20/HMX cocrystal in a nitrate ester. Herein, the aging experiment of a CL-20/HMX-based propellant was conducted to reveal the transformation of CL-20 and HMX under the effect of a nitrate ester, which contains nitroglycerin (NG) and 1,2,4-butanetriol trinitrate (BTTN). The aging properties of a CL-20-based propellant were also studied to exclude the effect of the phase transformation of ε-CL-20. Meanwhile, theoretical calculations were also carried out to study the cocrystal mechanism. The phase transformation of CL-20 at different temperatures, comparison between the CL-20/HMX cocrystal and mixture, as well as the CL-20/HMX/NG/BTTN mixture were investigated through molecular dynamics simulation.

## 2 Method

### 2.1 Propellant Samples

The CL-20/HMX-based propellant and CL-20-based propellant, which can be named CH0 and C0 samples were prepared by adopting the slurry-cast method. The propellant samples contained CL-20 and HMX (or CL-20), nitrate ester, aluminum powder, ammonium perchlorate (AP), GAP, and other additives (including a stabilizer and a curing agent). Ingredient percentages of the baseline formulation in weight are shown in Table 1.

**Table 1:** List of Sample Compositions (%)

	CL-20	HMX	NG	BTTN	Al	AP	GAP	other additives
CH0	20	32	8	8	17	5	5	5
C0	45	0	9	9	17	10	5	5

The aging process of propellants was carried out at a normal temperature and pressure. Aging samples were marked as CH1-6 and C1-6, which correspond to aging time of 30 days, 60 days, 90 days, 120 days, 150 days, and 180 days respectively.

### 2.2 Characterization Methods

#### 2.2.1 Scanning Electron Microscopy and Energy-Dispersive Spectroscopy

Different materials have different morphologies, and comparing the difference between propellant samples before and after aging can obtain the transformation of the component during the aging process. In this experiment, SEM was recorded using a Quanta FEG 600 (FEI) and the element mapping of the propellant was characterized by INCA IE 35V (Oxford).

#### 2.2.2 X-ray Diffraction

X-ray diffraction (XRD) is an effective approach for the analysis of a crystalline sample. In this experiment, XRD qualitative analysis is used to determine the cocrystallization process during aging by referring to XRD standard patterns of ε-CL-20, β-HMX, and CL-20/HMX cocrystals. XRD patterns are recorded using a PANalytical Empyrean with CuK_α_ radiation (λ = 1.5418 Å), equipped with a Vantec-1 detector and operated under the condition of 40 kV/mA. The 2θ ranged from 10 to 50° with a scanning step of 0.02°/0.1 s.^35^

#### 2.2.3 Fourier Transform Infrared Spectroscopy

FTIR measurement was performed using a model NEXUS 870 Fourier transform infrared spectrometer (Nicolet Instruments Co., USA). The experiment was carried out with a KBr pellet sample. IR spectra were in the range 4000–400 cm^–1^ with a resolution of 4 cm^–1^.

### 2.3 Computational Details

The transformation of the α-, β-, γ-, and ε-CL-20 crystal structure as well as the 10% defected samples was investigated through molecular dynamics simulations.

**Figure 1:**
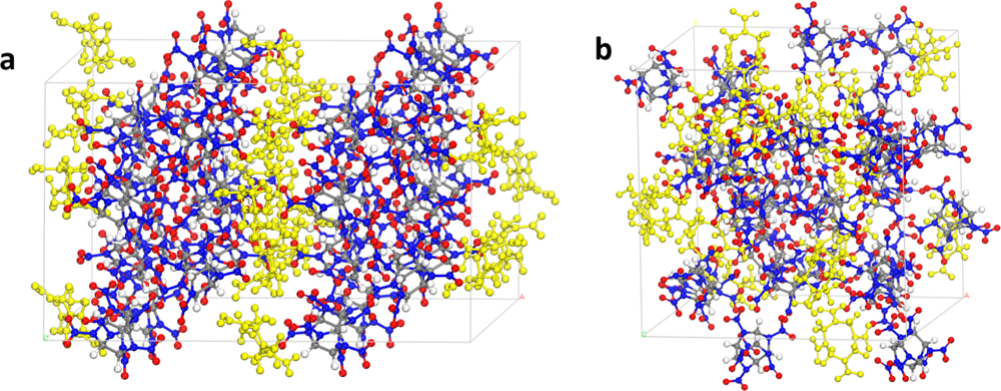
Equilibrium structure of the (a) CL-20/HMX cocrystal and (b) CL-20/HMX mixture (HMX in yellow).

The CL-20 crystal (α-, β-, γ-, and ε-phase), HMX crystal, and CL-20/HMX cocrystal structure are constructed according to cell parameters obtained from the Cambridge Crystallographic Data Center (CCDC) database. The models of the CL-20 crystal include perfect phases and defect phases including 10% vacancies. The structures of the cocrystal and mixture are built with a same molar ratio of 2:1 for ε-CL-20 and β-HMX, as shown in Figure 1. The mass fraction of the CL-20/HMX/NG/BTTN mixture is 20:32:8:8, which is the proportion used in a CL-20/HMX-based propellant sample. Meanwhile, CL-20/HMX/NG/BTTN mixtures with mass ratios of 20:32:4:4 and 20:32:0:0 were also constructed as control groups.

All calculations and simulations are conducted through applying Materials Studio (MS) 8.0 software. First, the structure of the model was optimized, molecular dynamics (MD) simulation was carried out according to the optimized stable configuration, the *NVT* ensemble was selected under the COMPASS force field,^36−38^ and Anderson was selected as the temperature control method.^39^ Considering the accuracy of the calculation results and configuration, the total simulation time of the calculation was set to 500 ps, the time step was 1.0 fs, and the most stable configuration in the last 100 ps was selected for follow-up calculation. Electrostatic and van der Waals interactions were calculated by Ewald and atom-based methods respectively, with a cutoff radius of 12.5 Å.

## 3 Results and Discussion

### 3.1 Experimental Study on Propellant Crystallization

**Figure 2:**
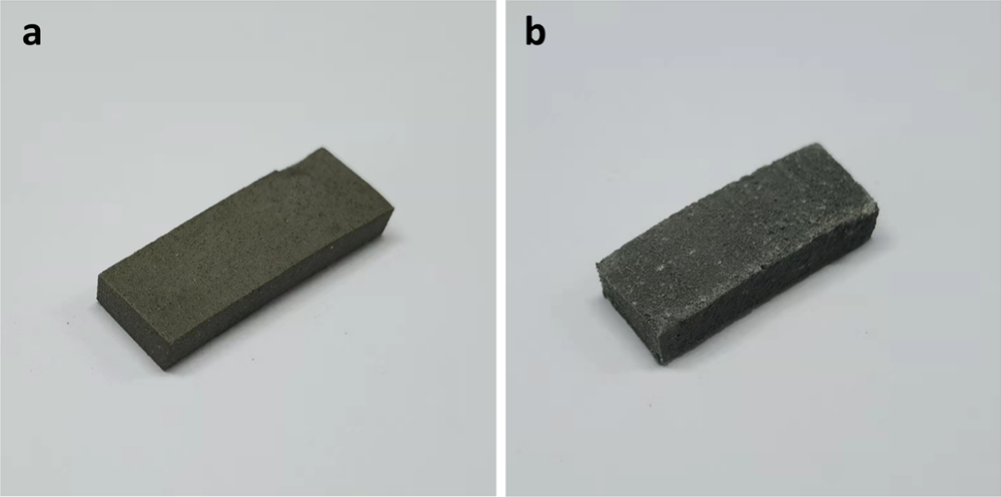
(a) CL-20-based propellant sample; (b) CL-20/HMX-based propellant sample.

The propellant samples used in the experiment include a CL-20-based propellant and a CL-20/HMX cocrystal-based propellant, as shown in Figure 2.

#### 3.1.1 SEM and Surface Element Analysis

**Figure 3:**
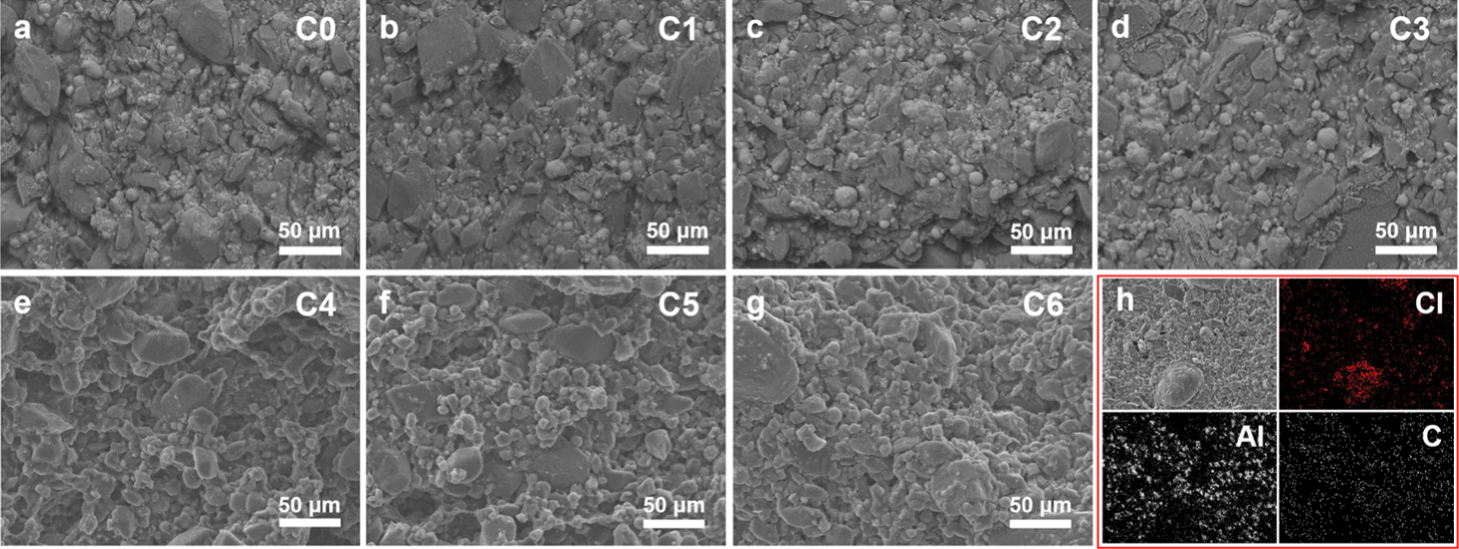
(a–g) SEM images of CL-20-based propellants; (h) element mapping images of sample C6.

**Figure 4:**
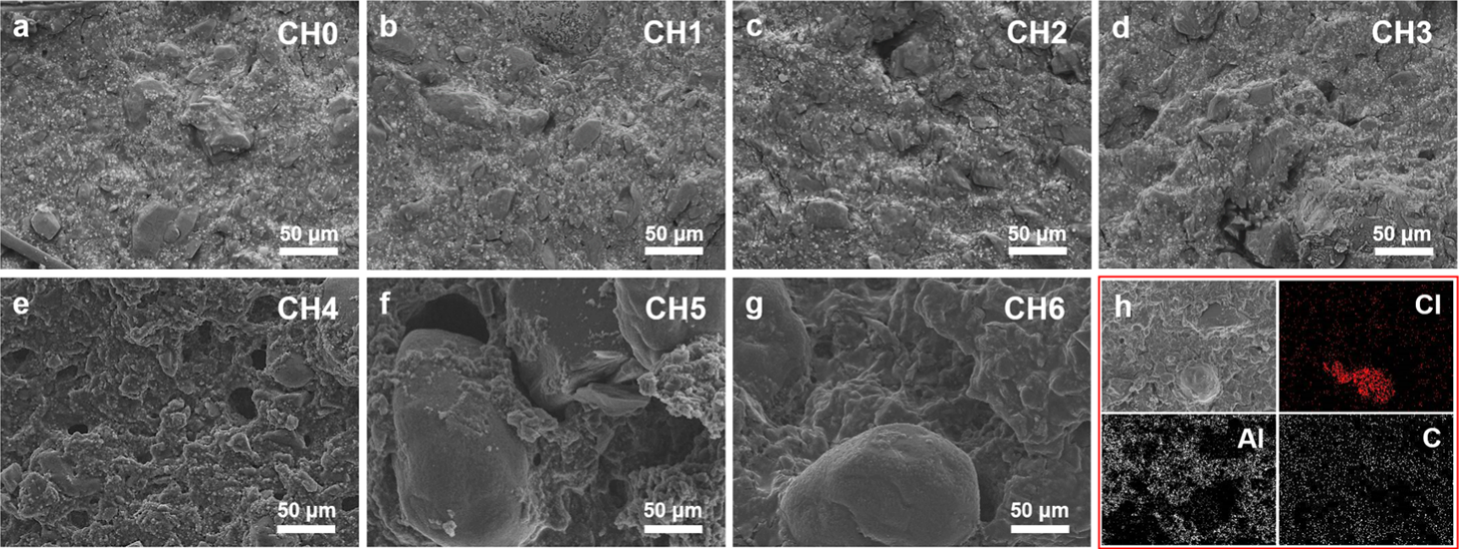
(a-g) SEM images of CL-20/HMX-based propellants; (h) element mapping images of sample CH6.

In order to understand the influence of cocrystallization of CL-20 and HMX on the morphology of a propellant, SEM-EDS analyses with some mapping images were conducted, and the results are shown in Figures 3 and 4.

Compared with the initial sample C0, the change of morphology of the CL-20-based propellant aging sample is not obvious at early aging. However, it can be seen from the SEM images of later aging samples that the outline is not as clear as that of the initial sample. This is because during the aging process of the propellant, CL-20 and other components that exist in the propellant in the form of crystals dissolve under the inducing effect of the solvent such as a nitrate ester. On the whole, the particles of each component are relatively tightly bonded to the matrix, and there is no obvious dehumidification and crystallization phenomenon. According to the information shown in Figure 3h, the red area is the element Cl, corresponding to AP particles. The uniform distribution of carbon means the matrix morphology is well maintained, and the agglomeration of Al is because Al metal exists in the form of powder. In general, the components are well-distributed in the propellant.

On the contrary, the aging sample of the CL-20/HMX-based propellant has a big change on morphology compared with the initial sample CH0, as Figure 4 shows. Similar to the CL-20-based propellant sample, the edges and corners tend to be rounded during the aging process, which is also caused by the partial dissolution of the components. In addition, in the CL-20/HMX-based propellant samples, larger pores appeared near the prismatic HMX crystals and spindle-shaped CL-20 crystals. Meanwhile, the combination between the crystal particles and the matrix is significantly worsened as the filler/matrix interface is destroyed. The mapping images show that there are some dark areas in the images of C and Al, which correspond to the pores caused by the cocrystal.

**Figure 5:**
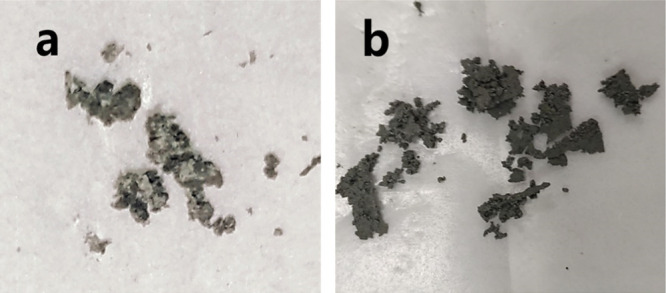
Digital photos of CH3 after mechanical stripping: (a) CL-20/HMX cocrystal phase; (b) binder continuous phase.

The energetic components of CH3 were extracted by a mechanical method. The digital photos of precipitated phase crystal particles and the continuous phase binder obtained after stripping are shown in Figure 5. It can be seen that crystallization occurs after aging, while the continuous phase binder is relatively flat, which indicates that CL-20 and HMX do agglomerate and form cocrystal particles during aging.

#### 3.1.2 XRD Analysis

**Figure 6:**
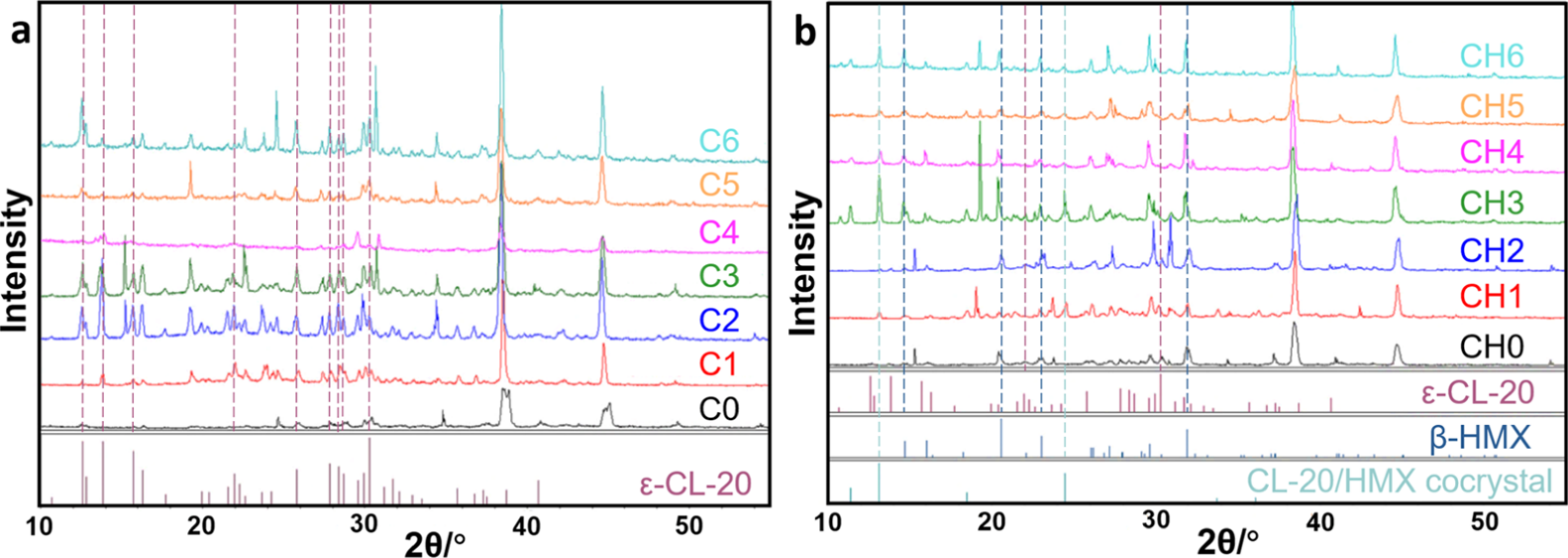
XRD patterns of (a) CL-20-based propellants; (b) CL-20/HMX-based propellants.

As seen in Figure 6a, the characteristic peaks of 10.736, 12.596, 12.831, 13.839, 15.701, 16.321, 25.811, 27.862, 28.487, and 30.361° correspond to ε-CL-20. The characteristic peaks of CL-20 in the initial sample C0 of the CL-20-based propellant are not particularly obvious. With the increase of aging time, the characteristic peaks of ε-CL-20 in each sample increase obviously, and no crystal transformation is found.

It can be seen in Figure 6b that the characteristic peaks of 10.736, 12.596, 12.831, 13.839, 15.701, 16.321, 25.811, 27.862, 28.487, and 30.361° correspond to ε-CL-20. The characteristic peaks of 14.766, 16.139, 18.380, 20.635, 22.252, 23.150, 26.245, 27.359, 28.120, 29.736, 31.978, 37.221, and 41.337° correspond to β-HMX. It can be figured out that the diffraction pattern of CH0 is a simple composition of the peaks of ε-CL-20 and β-HMX, in which the characteristic peaks of β-HMX are stronger. With the increasing of aging time, the peak intensities of ε-CL-20 and β-HMX increased, and the peaks of β-HMX are still higher than that of ε-CL-20, indicating that CL-20 and HMX both go through crystallization during the aging process. Meanwhile, new characteristic peaks appear in the diffraction patterns of sample CH4-6, located at 11.4, 13.2, 18.6, 24.5, 33.7, and 36.2°. These new peaks are consistent with the characteristic peaks of CL-20/HMX cocrystals reported in the literature,^27^ indicating the cocrystallization of the raw materials. The characteristic peaks of ε-CL-20 gradually weaken in the patterns of these samples and even disappeared. However, the peaks of β-HMX remain, and the peaks of the CL-20/HMX cocrystal appear at the same time. It is because CL-20 and HMX generally cocrystallized in a molar ratio of 2:1. During aging, CL-20 was consumed because of its participation in cocrystallization, while HMX can still be detected due to its rapid crystallization rate and less molar ratio in the cocrystal. From the above analysis, it can be concluded that when ε-CL-20 and β-HMX coexist in propellants, they tend to be cocrystals rather than blends.

#### 3.1.3 FTIR Analysis

**Figure 7:**
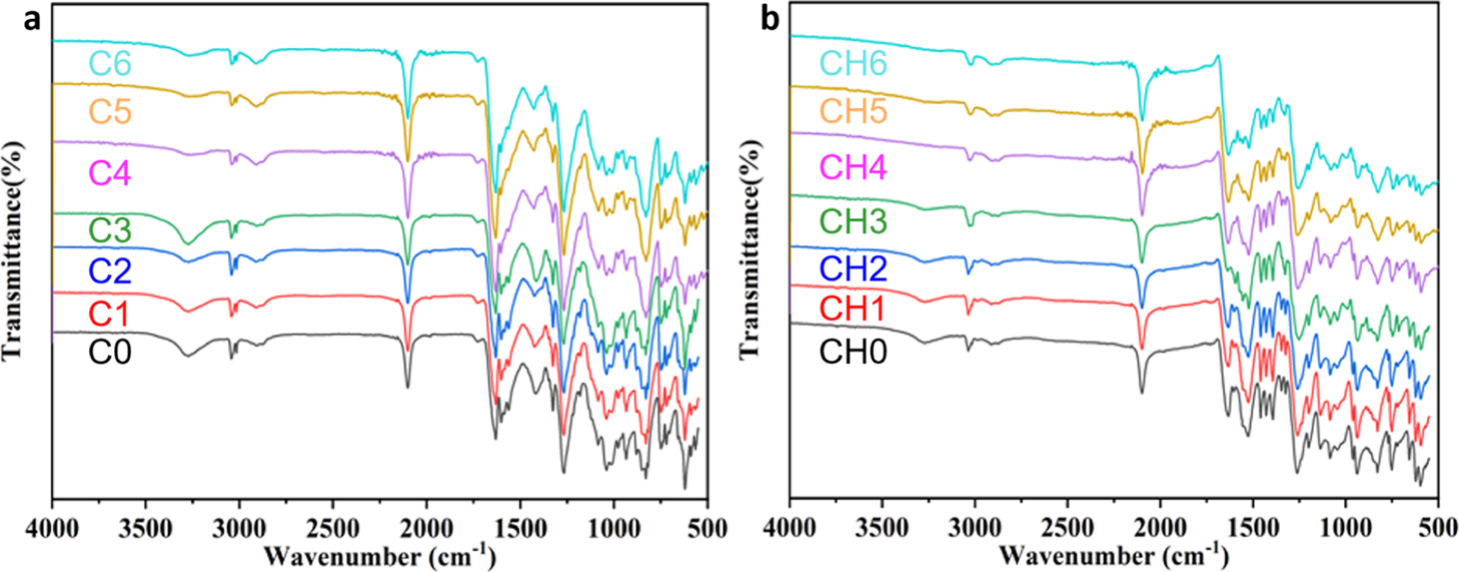
FTIR spectra of (a) CL-20-based propellants and (b) CL-20/HMX-based propellants.

As shown in Figure 7a, there is an absorption peak at 2900–3036 cm^–1^ in the FTIR spectra of the initial sample C0 of the CL-20-based propellant, which is the stretching vibration of −CH_2_– in ε-CL-20. The absorption peak at 2100 cm^–1^ is the asymmetric stretching vibration of −N_3_ azide in GAP. It can be found that the FTIR spectra of the aging sample C1-6 are basically consistent with those of the initial sample, indicating that there is no chemical reaction before and after aging. However, the absorption peak at 1563 cm^–1^, which corresponds to −NO_2_ of the spectra of aging samples weaken with the increasing of aging time, indicating that the nitro group might decompose during the aging process.

The FTIR spectra of sample CH0-6 are shown in Figure 7b. The absorption peak at 1524 cm^–1^, which is the characteristic absorption peak of HMX, almost disappears in the spectra of sample CH6, indicating that the component of HMX has changed. The peak at 1634 cm^–1^ is the asymmetric vibration absorption peak of −NO_2_ in ε-CL-20. It is worth noting that this peak obviously gets weakened with the increase of aging time. Moreover, peaks at 3035 cm^–1^, which represent the −CH_2_– stretching of the carbon chain, shifted to 3013 cm^–1^ at samples CH4-6. It is because hydrogen bonds formed between −NO_2_ of CL-20 and −CH_2_- of HMX as a result of the CL-20/HMX cocrystal.^25,40^ Combined with the above judgment, C–H···O hydrogen bonds are formed to a certain extent. This is also proof of cocrystal formation between CL-20 and HMX.

### 3.2 Theoretical Study on Propellant Crystallization

#### 3.2.1 Stability of Different Phases of CL-20 Crystal

In order to explore the phase transformation of CL-20 in the solid propellant aging process, the stability of each crystal phase of the CL-20 crystal was analyzed, and four main crystal phases of CL-20 (α, β, γ, and ε) were constructed. The models include perfect phases and defect phases including 10% vacancies. After molecular dynamics optimization at different temperatures, the crystal transformation rate was obtained using the XRD method.

**Figure 8:**
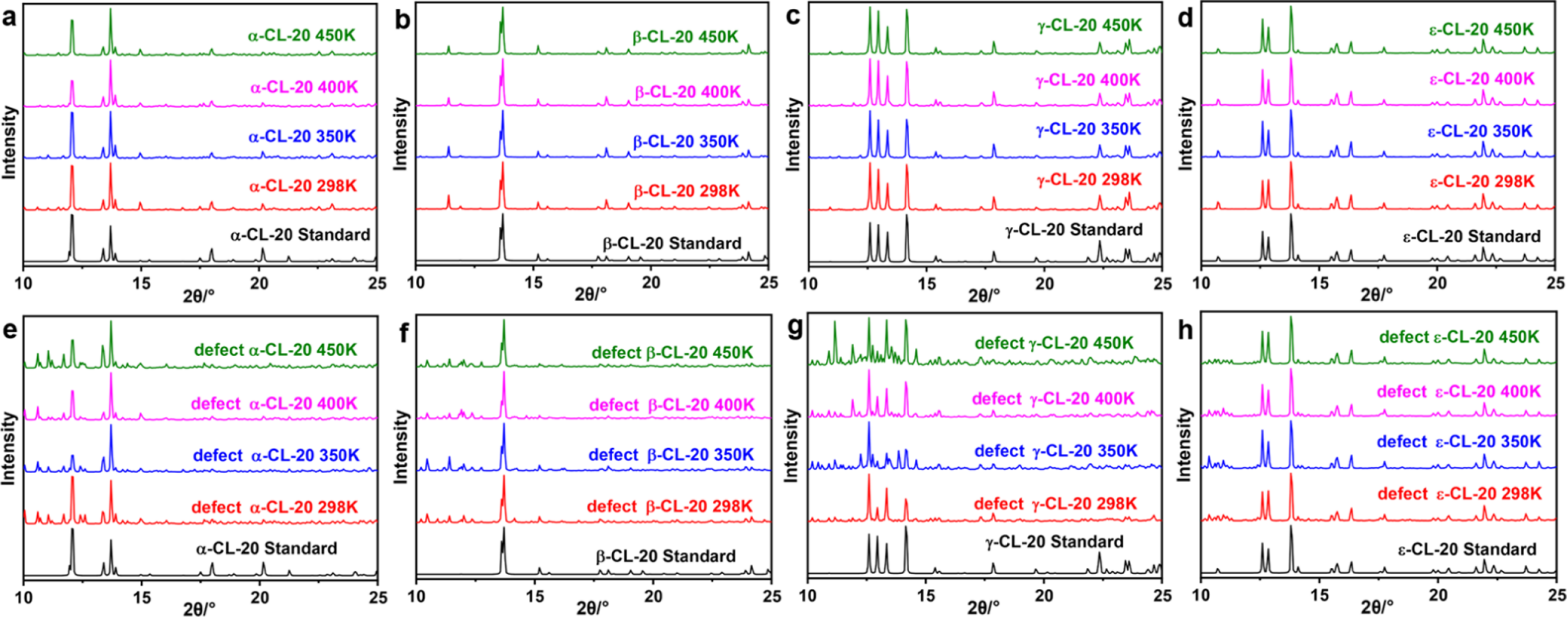
XRD pattern of (a–d) perfect CL-20 crystals and (e–h) defect CL-20 crystals.

**Figure 9:**
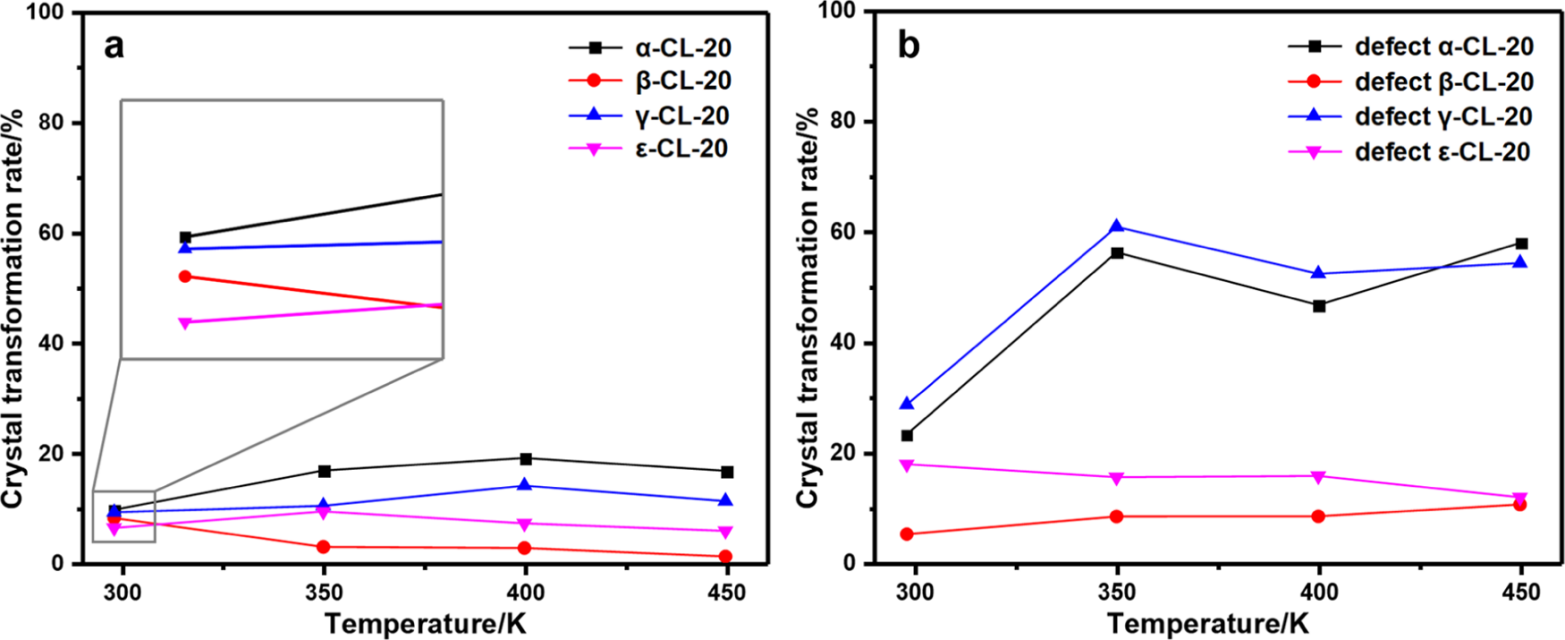
Crystal transformation rate of (a) perfect CL-20 and (b) defect CL-20.

XRD patterns of perfect and defect CL-20 crystals at different temperatures were obtained, as shown in Figure 8. Due to the little change during crystal transformation, it is difficult to obtain detailed crystal transformation data by direct analysis of the XRD pattern. Therefore, we quantitatively analyzed the XRD data, obtained the content of the corresponding form in each crystal structure, and the crystal transformation rate is shown in the Figure 9. It can be seen that the crystal transformation rate of defect CL-20 is higher than that of perfect CL-20, which is because of the vacancy in the crystal. Whether in the perfect crystal or the defect crystal, the crystal transformation rates of the α-phase and the γ-phase are higher than those of the β-phase and the ε-phase. With the increase of temperature, the change of crystal transformation rate is relatively gentle. It should be noted that the crystal phase after transformation is not necessarily one of the α-, β-, γ- or ε-phase. Some of them are in the intermediate state of the four crystal forms due to different inversion angles.

The result shows that ε-CL-20 is relatively stable during the temperature changes. Among these four phases, the crystal transformation rate of the ε-phase is just a little higher than that of the β-phase. However, the ε-phase is the most stable at room temperature, indicating that ε-CL-20 tends to be stable in the aging process rather than transfer to other crystal phases.

#### 3.2.2 Comparison of CL-20/HMX Cocrystal and Mixture

##### 3.2.2.1 Binding Energy

In order to explore the difference between the CL-20/HMX cocrystal and the mixture, the structures of the cocrystal and the mixture are built with the same molar ratio. First of all, we calculate the binding energy *E*_bind_ of the CL-20/HMX cocrystal and the mixture at room temperature equilibrium. The binding energy is defined as the negative value of the intermolecular interaction energy (*E*_inter_), which can well reflect the compatibility of the two components blended with each other. The intermolecular interaction energy can be calculated by the total energies of the whole system and individual component energy in the system. As such, the binding energy between CL-20 and HMX can be expressed as follows
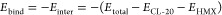
where *E*_CL-20_, *E*_HMX_, and *E*_total_ are energies of CL-20, HMX, and the whole system, respectively, which are tabulated in Table 2. *E*_bind_ of the cocrystal and the mixture are calculated to be 907.65 kcal/mol and 764.82 kcal/mol, respectively. The larger binding energy means stronger intermolecular binding, indicating that CL-20 and HMX tend to form cocrystals thermodynamically.

**Table 2:** Binding Energies of the CL-20/HMX Cocrystal and the Mixture (kcal/mol)^a^

	*E*_total_	*E*_CL-20_	*E*_HMX_	*E*_bind_
cocrystal	–13346.04	–9081.40	–3356.99	907.65
mixture	–13183.93	–9067.37	–3351.75	764.82

a Radial distribution function analysis.

The radial distribution function (RDF) is a useful physical tool because it provides insights into a material structure by a measure of the local spatial ordering, which gives a measure of the probability density *g*(*r*) of finding an atom at some distance from a reference atom. Generally, the interaction distance range (*r*) for the hydrogen bond is 2.0–3.1 Å and for strong van der Waals, it is 3.1–5.0 Å. When *r* is farther than 5.0 Å, the van der Waals is very weak.^41^

**Figure 10:**
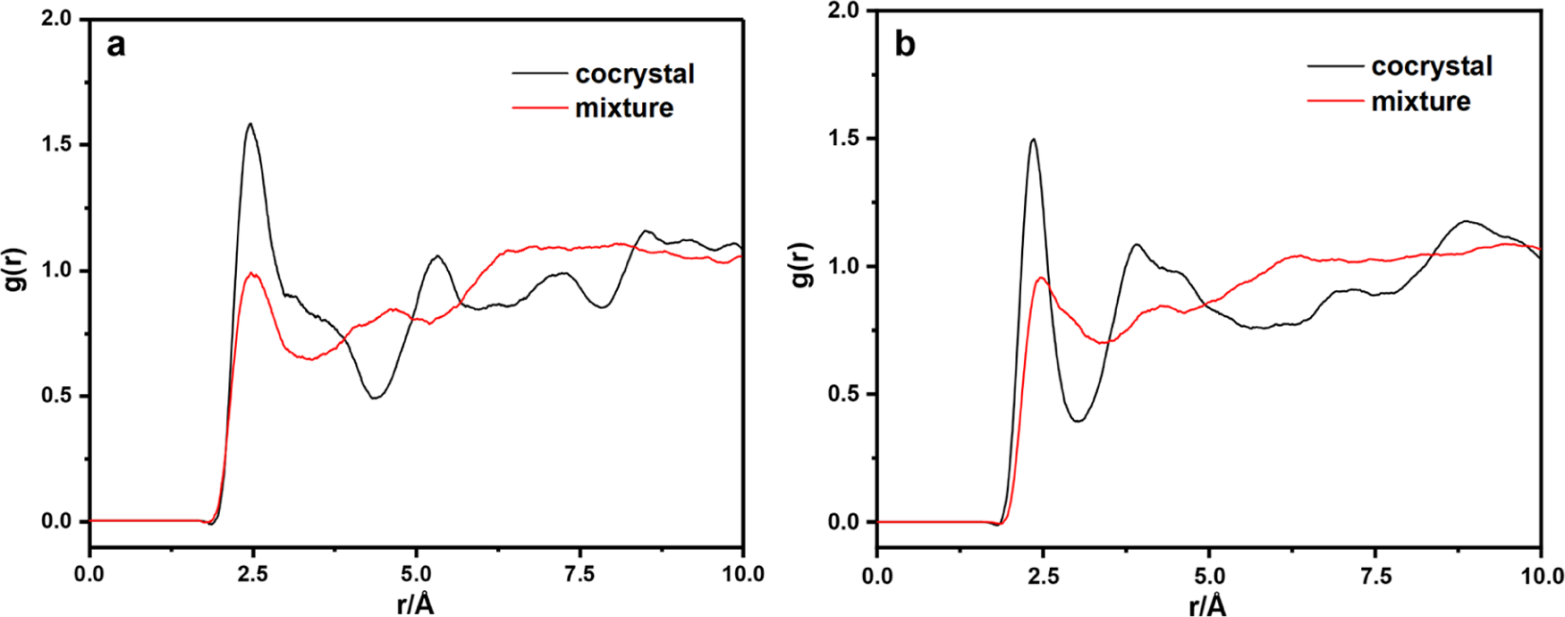
RDF for (a) H(1)···O(2) and (b) H(2)···O(1) in the CL-20/HMX cocrystal and the mixture.

In this paper, H···O hydrogen bonds are considered between CL-20 and HMX molecules. H and O atoms in CL-20 molecules were denoted as H(1) and O(1), and those in HMX molecules were named as H(2) and O(2). Figure 10a,b shows the RDF for H(1)–O(2) and H(2)–O(1), respectively. The first peaks of the curves are all located at about 2.5 Å, which means that hydrogen bonds exist in H(1)···O(2) and H(2)···O(1) pairs. It can be found that the value of g(r) of the cocrystal is much larger than that of the mixture. This implies that the interaction of the two atom pairs between CL-20 and HMX in the cocrystal is stronger than that in the mixture, which is consistent with the conclusion that *E*_bind_ of the CL-20/HMX cocrystal is much larger than that of the mixture.

##### 3.2.2.2 Mechanical Properties

Mechanical properties are some of the most important properties of energetic materials due to their relationship with material preparation, storage, transportation, and usage. The elastic modulus is an indicator of the material stiffness and a measurement of the material resistance to elastic deformation. It is known from the elastic mechanics that the relationship between stress and strain can be expressed by the generalized Hooke’s law as follows
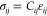
where *C*_*ij*_ (*i*, *j* = 1, 2, ..., 6) are the elastic constants matrix of the materials, and the elastic constants matrix satisfies the relationship *C*_*ij*_ = *C*_*ji*_ because of the existence of strain energy. Hence, at most, 21 independent elastic constants are required to describe the stress–strain relationship for any material. The tensile modulus (*E*), bulk modulus (*K*), shear modulus (*G*), and Poisson’s ratio (ν) can be calculated as follows
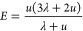

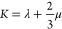

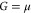

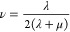

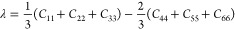

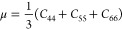
where λ and μ are the lame constants.^42−44^

In this paper, the mechanical properties of the models were measured through the constant-strain approach implemented in a Materials Studio, which are shown in Table 3.

**Table 3:** Mechanical Properties of Different Models (GPa)

models	tensile modulus (*E*)	bulk modulus (*K*)	shear modulus (*G*)	Poisson’s ratio (ν)
ε-CL-20	14.41	13.02	5.48	0.32
β-HMX	14.24	6.83	6.18	0.15
cocrystal	11.81	9.63	4.56	0.30
mixture	5.46	7.77	1.97	0.38

In general, the bulk modulus, shear modulus, and the tensile modulus are often used to evaluate the rigidity and ability to resist the elastic deformation of the material. It can be concluded from the results that the CL-20/HMX mixture has poor mechanical properties, low stiffness, and is easy to break under the action of an external force. However, the tensile modulus and shear modulus of the CL-20/HMX cocrystal are both relatively low, indicating that the cocrystal has better flexibility and variability. The other values of mechanical properties of the cocrystal are between those of ε-CL-20 and β-HMX. In short, the comprehensive mechanical properties of the CL-20/HMX cocrystal are better than the others. This is because the stronger hydrogen bond in the cocrystal makes the internal molecules of the system more closely bound, and the mechanical properties have been improved as a result.

#### 3.2.3 Analysis of CL-20/HMX/NG/BTTN Mixture

In order to better simulate the environment of CL-20 and HMX in the propellant, we constructed the CL-20/HMX/NG/BTTN mixed model, with a mass ratio of 20:32:8:8 and 20:32:4:4 which can be named as mixture I and mixture II, respectively. In addition, we built a CL-20/HMX mixed model with a mass ratio of 20:32 as a blank comparison.

**Figure 11:**
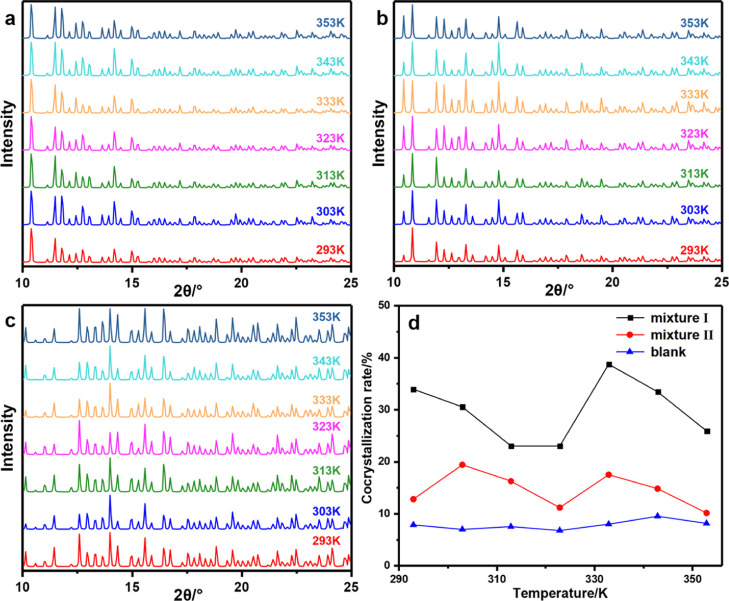
XRD pattern of (a) mixture I, (b) mixture II, and (c) blank; (d) Curves of cocrystallization rate.

We conduct the comparative analysis with the XRD of CL-20/HMX cocrystal and calculate the percentage in forming a CL-20/HMX cocrystal, namely, cocrystallization rate, of mixtures I and II and the blank mixture at different temperatures, as shown in Figure 11. It can be seen that the cocrystallization rate has a nonlinear relationship with temperature. It should be noted that at the same temperature, the cocrystallization rates of mixtures I and II are higher than that of the blank mixture, which means that the nitrate solvent will promote the cocrystallization of CL-20 and HMX. Moreover, the cocrystallization rate is related to the amount of nitrate solvent; the more the nitrate solvent, the more it can promote cocrystallization. It is because CL-20 and HMX can dissolve in the nitrate solvent, and the interaction between CL-20 and HMX molecules will cause cocrystal formation. This result well explains the cocrystal phenomenon in the propellant aging process and provides a good theoretical basis for the experimental study of increasing the percentage of the CL-20/HMX cocrystal.

### 3.3 Discussion Based on Experiments and Calculations

**Figure 12:**
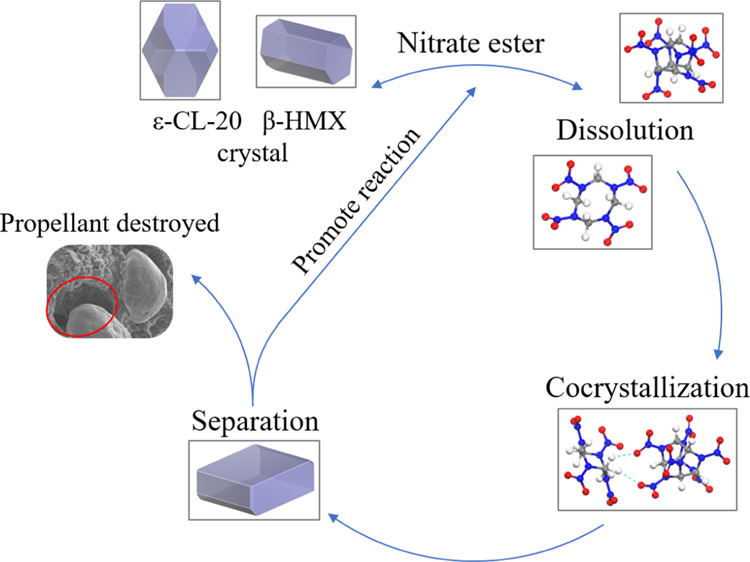
Cocrystal mechanism of CL-20 and HMX in the nitrate ester of a propellant.

Based on the theoretical calculations and experimental results, it can be seen that ε-CL-20 and β-HMX crystals undergo a dissolution–cocrystallization process under the inducing effect of a nitrate ester in the propellant. When CL-20 exists in the nitrate solvent alone, it will reach saturation very soon because of the limited solubility. Therefore, the changes of morphology of the CL-20-based propellant in only the edges and corners tend to be rounded. However, when CL-20 and HMX exist in nitrate in the propellant at the same time, molecules will separate out from the matrix in the form of cocrystals because of the cocrystallization between CL-20 and HMX. The dissolution equilibrium is destroyed and the chemical reaction moves to the right, causing more ε-CL-20 and β-HMX crystals to turn into molecules dissolved in the solvent and separate out as cocrystals. A “dissolution–cocrystallization–separation–dissolution” cycle is formed, leading to a large consumption of ε-CL-20 and β-HMX crystals, as shown in Figure 12. In this process, because the sites’ dissolution of original crystals and the separation of cocrystals are not necessarily the same, plus the size of the cocrystal is smaller than those of ε-CL-20 and β-HMX crystals, some pores will occur in the propellant after aging, resulting in the decrease in the density of the propellant, deterioration of the mechanical properties, and loss of use value.

It should be noted that when CL-20 and HMX crystals exist in any solvent in which both CL-20 and HMX can dissolve in at same time, they tend to form cocrystals rather than coexist as molecules.

## 4 Conclusions

In this work, we mainly studied the cocrystallization mechanism in a solid propellant under the inducing effect of a nitrate ester through theoretical calculations and experiments. The major findings can be summarized as follows:

(1) Aging experiments show that the CL-20/HMX cocrystal occurs in the CL-20/HMX-based propellant, leading to pores and dehumidification in the propellant, while there is no such phenomenon in the CL-20-based propellant.
(2) Among the four crystalline phases of CL-20, the ε-phase is the most stable at room temperature and is relatively stable during temperature changes, indicating that ε-CL-20 is suitable for application in a solid propellant.
(3) CL-20/HMX cocrystal has better stability and mechanical properties than the CL-20/HMX mixture because of the intermolecular hydrogen bonds. Moreover, more nitrate ester will promote the nucleation of the CL-20/HMX cocrystal.
(4) When CL-20 and HMX exist in nitrate in the propellant at the same time, molecules will separate out from the matrix in the form of cocrystals because of the cocrystallization between CL-20 and HMX. A “dissolution–cocrystallization–separation–dissolution” cycle is formed, leading to a large consumption of ε-CL-20 and β-HMX crystals, resulting in the decrease in the density of the propellant, deterioration of the mechanical properties, and loss of the use value.

## Supporting Information Available

Additional experimental data (SEM images) of raw CL-20 and HMX crystals and the peak area ratio of FTIR in CL-20-based propellants and CL-20/HMX-based propellants (PDF)
