# Supplementary material for: Study on the Cocrystallization Mechanism of CL-20/HMX in a Propellant Aging Process through Theoretical Calculations and Experiments
Source: ACS Omega. 2022 Feb 18;7(8):7361–9. doi: 10.1021/acsomega.1c07328 (PMC8892642; doi:10.1021/acsomega.1c07328)
Supplement: Supplementary file 1 — ao1c07328_si_001.pdf [file ao1c07328_si_001.pdf]

Supplementary Information

Study on cocrystallization mechanism of CL-20/HMX in propellant aging process through theoretical calculation and experiment

Xitong Zhao [a], Xiaolong Fu [a], Guanglong Zhang [b], Xiangyang Liu [b*], Xuezhong Fan [a*]

[a] *Xi'an Modern Chemistry Research Institute, Xi'an 710065, Shaanxi, China*

[b] *School of Aerospace Engineering, Beijing Institute of Technology, Beijing 100081, Beijing, China*

* *Corresponding Author: E-mail address: xuezhongfan@126.com (Xuezhong Fan). liuxy@bit.edu.cn (Xiangyang Liu).*

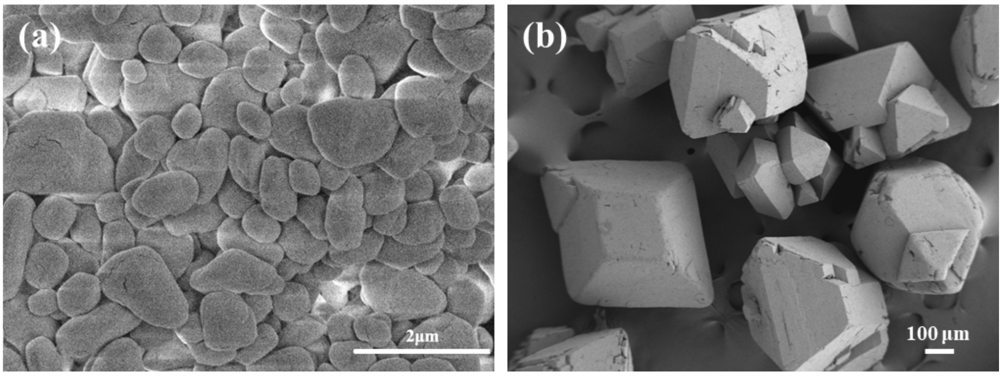

Figure S1 SEM images of (a) CL-20; (b) HMX

Table S1 The peak area ratio of FTIR in CL-20 based propellants (%)

	C0	C1	C2	C3	C4	C5	C6
1563 cm^{-1}	18.49	11.01	13.42	8.25	7.62	7.52	8.86

Table S2 The peak area ratio of FTIR in CL-20/HMX based propellants (%)

	CH0	CH1	CH2	CH3	CH4	CH5	CH6
1524 cm^{-1}	21.70	20.88	20.69	16.45	14.08	12.76	7.75
1634 cm^{-1}	12.65	8.04	8.41	6.37	7.27	8.01	8.36
